# DEGRONOPEDIA: a web server for proteome-wide inspection of degrons

**DOI:** 10.1093/nar/gkae238

**Published:** 2024-04-03

**Authors:** Natalia A Szulc, Filip Stefaniak, Małgorzata Piechota, Anna Soszyńska, Gabriela Piórkowska, Andrea Cappannini, Janusz M Bujnicki, Chiara Maniaci, Wojciech Pokrzywa

**Affiliations:** Laboratory of Protein Metabolism, International Institute of Molecular and Cell Biology in Warsaw, 4 Ks. Trojdena Str., 02-109 Warsaw, Poland; Laboratory of Bioinformatics and Protein Engineering, International Institute of Molecular and Cell Biology in Warsaw, 4 Ks. Trojdena Str., 02-109 Warsaw, Poland; Laboratory of Protein Metabolism, International Institute of Molecular and Cell Biology in Warsaw, 4 Ks. Trojdena Str., 02-109 Warsaw, Poland; Laboratory of Protein Metabolism, International Institute of Molecular and Cell Biology in Warsaw, 4 Ks. Trojdena Str., 02-109 Warsaw, Poland; Laboratory of Protein Metabolism, International Institute of Molecular and Cell Biology in Warsaw, 4 Ks. Trojdena Str., 02-109 Warsaw, Poland; Laboratory of Bioinformatics and Protein Engineering, International Institute of Molecular and Cell Biology in Warsaw, 4 Ks. Trojdena Str., 02-109 Warsaw, Poland; Laboratory of Bioinformatics and Protein Engineering, International Institute of Molecular and Cell Biology in Warsaw, 4 Ks. Trojdena Str., 02-109 Warsaw, Poland; Medical Research Council (MRC) Protein Phosphorylation and Ubiquitylation Unit, School of Life Sciences, University of Dundee, Dow Street, Dundee DD1 5EH, UK; Laboratory of Protein Metabolism, International Institute of Molecular and Cell Biology in Warsaw, 4 Ks. Trojdena Str., 02-109 Warsaw, Poland

## Abstract

E3 ubiquitin ligases recognize substrates through their short linear motifs termed degrons. While degron-signaling has been a subject of extensive study, resources for its systematic screening are limited. To bridge this gap, we developed DEGRONOPEDIA, a web server that searches for degrons and maps them to nearby residues that can undergo ubiquitination and disordered regions, which may act as protein unfolding seeds. Along with an evolutionary assessment of degron conservation, the server also reports on post-translational modifications and mutations that may modulate degron availability. Acknowledging the prevalence of degrons at protein termini, DEGRONOPEDIA incorporates machine learning to assess N-/C-terminal stability, supplemented by simulations of proteolysis to identify degrons in newly formed termini. An experimental validation of a predicted C-terminal destabilizing motif, coupled with the confirmation of a post-proteolytic degron in another case, exemplifies its practical application. DEGRONOPEDIA can be freely accessed at degronopedia.com.

## Graphical Abstract

**Graphical Abstract:**
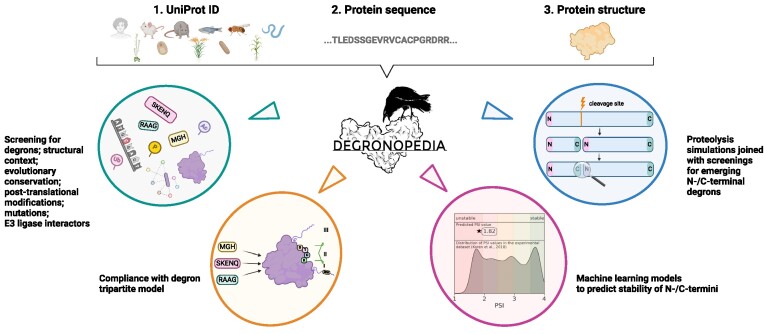

## Introduction

In the ubiquitin-proteasome system (UPS), E3 ubiquitin ligases are critical for tagging proteins for degradation, primarily attaching ubiquitin to their lysine residues (1,2), with emerging evidence indicating cysteine, serine, and threonine residues also acting as ubiquitination sites (3,4). Proteins are targeted for degradation through degrons, typically short linear motifs found in disordered regions, which can be constitutive or conditional, the latter emerging from post-translational modifications (PTMs) (5). It is important to emphasize that degrons do not solely exist as linear sequences. For example, numerous proteins possess zinc fingers and a structurally conserved β-hairpin loop, potentially serving as structural degrons (6,7). Degrons are not restricted in location but those at the amino or carboxyl termini, integral to the N- or C-degron pathways, have been extensively studied (5,8–14).

Recent research suggests that ubiquitination and subsequent degradation depend not just on degron recognition. The tripartite degron model proposed by Guharoy *et al.* introduces secondary degrons, comprising lysine residues near the primary degron, and tertiary degrons, which are flexible, intrinsically disordered regions (IDR) nearby, facilitating protein unfolding before proteasomal entry (15). The secondary and tertiary degrons are suggested to play subsidiary roles that affect ubiquitin-signaling. Deficiency of one of the elements of the tripartite degron model, e.g. an IDR near a ubiquitinated lysine, can result in non-proteolytic ubiquitination functions (15).

While research on degron motifs and their physiological roles is growing, there are few bioinformatics tools for degron motif screening and analysis. The APC/C degron repository (16) offers insights into APC/C degron sequence determinants, including information on disordered regions and post-translational modifications. The Eukaryotic Linear Motif (ELM) resource (17) allows detection of 32 degron motifs in proteins, providing structural context. An interactive web table based on ELM data and further manual curation, released in 2017, lists degron motifs (https://dosztanyi.web.elte.hu/CANCER/DEGRON/TP.html) (18). Additionally, deep learning models like Degpred (19) and deepDegron (20) have been developed for predicting degrons and their disruption by mutations, respectively. However, deepDegron, which predicts the potential for a protein sequence to contain a degron, is a stand-alone tool requiring specific inputs, like mutations in a Mutation Annotation Format file. Degpred, while limited to the human proteome, does not provide PTMs information that can affect degron functionality and mainly predicts internal degrons based on sequence alone, without considering their tertiary protein structure. DegronMD (21), a new resource, predicts and maps human internal degrons as well; however, its training was based on a limited dataset of 23 internal degron motifs from the ELM database. To our knowledge, no existing resource comprehensively compiles all known degron motifs for thorough screening in query proteins, covering both termini and internal areas, nor provides detailed data on degron site context, such as in the proposed tripartite degron model, or offer options for customized calculations.

Addressing these complexities, we introduce DEGRONOPEDIA, a web server for identifying and analyzing degron motifs in proteins. It provides detailed analyses of sequence, spatial, and tripartite model context, including PTMs and mutations, and features pre-calculated multiple sequence alignments (MSA) for evolutionary insights, with an option for custom uploads. Moreover, DEGRONOPEDIA allows for the simulation of sequence cleavage and identification of potential N-/C-degrons post-proteolysis. It also employs machine learning (ML) models to predict protein terminal stability, trained on datasets from high-throughput human proteome terminal stability studies.

Our successful identification of a degron at the C-terminus of FBXL15, as well as confirming the presence of a predicted degron in SDE2 post-proteolysis, demonstrates DEGRONOPEDIA usefulness for researchers in many areas. Our server offers a user-friendly interface, comprehensive tutorial, documentation and introductory video, making it accessible to a wide range of users. To the best of our knowledge, DEGRONOPEDIA is unique in its scope by providing the most current and extensive collection of degrons, coupled with detailed analyses of their molecular characteristics, ML-based predictions, and simulations of protein cleavage.

## Materials and methods

### Inputs

Three input types can be used for querying DEGRONOPEDIA: (i) a UniProt ID of a protein from the reference proteome (according to the UniProt database (22)) of one of the selected model organisms—*H. sapiens, M. musculus, R. norvegicus, D. rerio, D. melanogaster, C. elegans, S. cerevisiae, S. pombe, A. thaliana, O. sativa* or *Z. mays*, (ii) a protein sequence in the FASTA format and (iii) a protein structure (either a protein model or a structure obtained in experimental studies) in the PDB format; any submitted PDB file must contain a protein monomer with only one model and one chain with continuous numbering starting from 1 and not exceeding 5 MB in size. Regardless of the input type, the query protein must be below 40 000 canonical amino acids.

### Outputs

**Figure 1.**
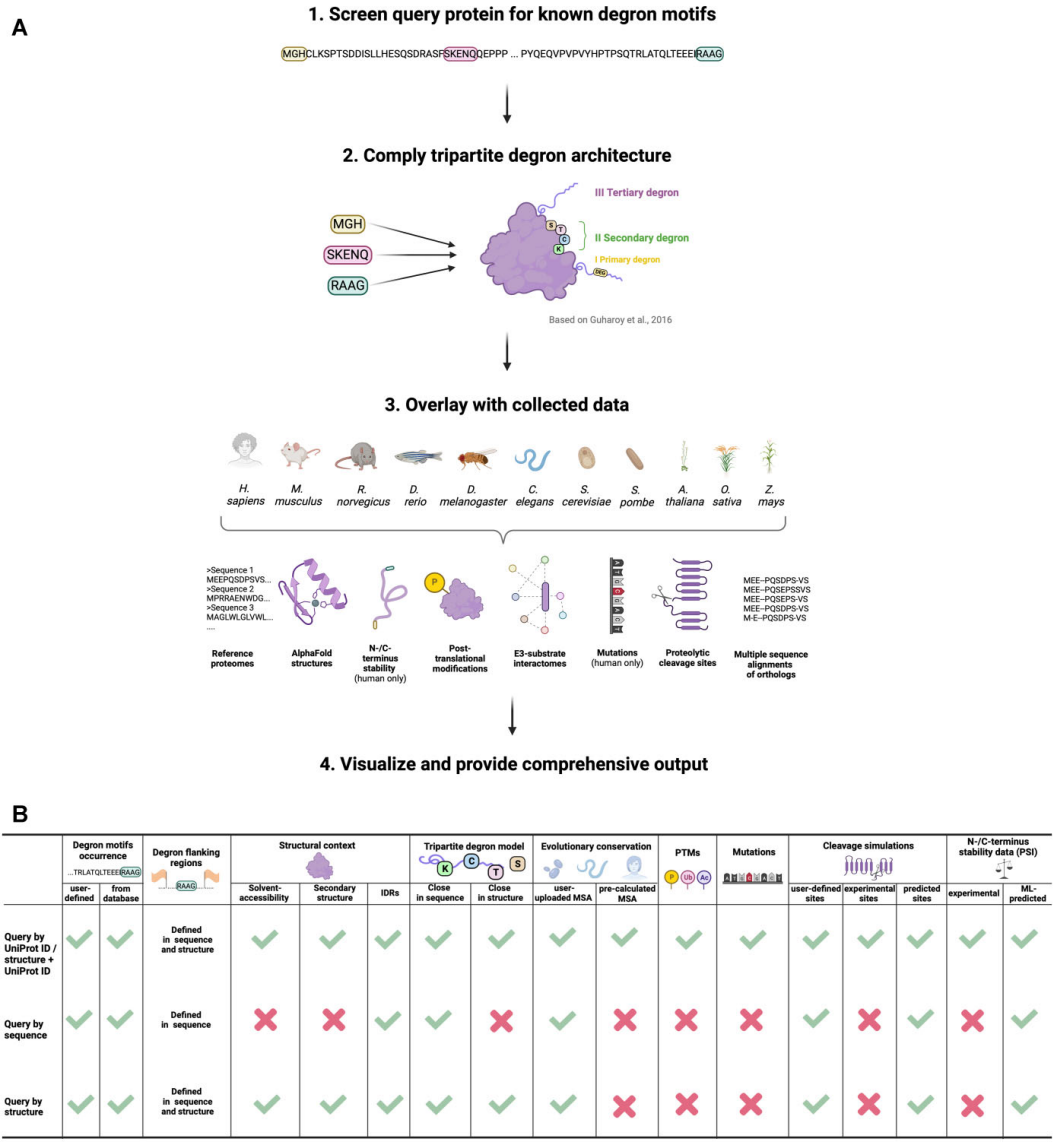
DEGRONOPEDIA implementation details. (**A**) Operational scheme of server functioning when querying by UniProt ID. (**B**) Comparison of the output information obtained upon different query types.

DEGRONOPEDIA output varies depending on the type of input: a query by UniProt ID delivers the most comprehensive degron information, including a full tripartite model, N-/C-terminus stability expressed as Protein Stability Index (PSI) based on the Global Protein Stability (GPS) studies (11,12) (experimental and/or predicted values), data on evolutionary conservation, structural context (derived from corresponding AlphaFold2 model from the AlphaFold Protein Structure database (23,24)), PTMs, mutations, physiologically-relevant proteolytic cleavage sites, and E3 ubiquitin ligase interactors. In contrast, a query by FASTA provides basic screening output, missing experimental data. A query by structure offers a moderate level of detail with the tripartite model but lacks experimental data. Lastly, combining structure with UniProt ID yields the same extensive information as a UniProt ID query, except that the structural data, i.e. secondary structure, solvent-accessibility, IDRs, are based on the uploaded structure. Irrespective of the query type, users can download an xlsx file with all the results, organized into distinct sheets. The summary of DEGRONOPEDIA workflow and the granularity of the output depending on the input type is presented in Figure 1.

### Implementation

#### Datasets

**Degron motifs**. We compiled over 1800 degron motifs (5,11,12,17,25–31). Each motif was defined as either N-terminus, C-terminus or internal, regarding its location. The list of degron motifs is continuously updated and can be downloaded from the server.

**Reference proteomes and structural data**. Reference proteomes for *H. sapiens, M. musculus, R. norvegicus, D. rerio, D. melanogaster, C. elegans, S. cerevisiae, S. pombe, A. thaliana, O. sativa* and *Z. mays* were obtained from the UniProt database (IDs: UP000005640, UP000000589, UP000002494, UP000000437, UP000000803, UP000001940, UP000002311, UP000002485, UP000006548, P000059680, UP000007305, respectively), along with structural models from the AlphaFold Protein Structure database. In cases of proteins exceeding 2700 amino acids, an in-house script using Biopython (32) merged AlphaFold2 model fragments into single PDB files.

**Orthologs data**. Pre-calculated MSAs of predicted orthologs were obtained from the eggNOG5 database (33) at various taxonomic levels (Supplementary Table S1).

**N-/C-termini stability data**. The N-/C-termini stability data were obtained from the GPS assays (11,12). These data cover the stability of N-/C-terminal 23-mers of 48251 (variants with N-terminal methionine and without it) and 22564 human proteins, respectively, and are represented as PSI, where its lowest values indicate the most unstable, thus possibly containing a degron motif, peptides.

**Post-translational modification data**. The PTMs datasets were obtained from the iPTMNet (34), PhosphoSitePlus (35), Plant PTM Viewer (36), and the PLMD (37) databases as well as from the literature, where we manually compiled datasets of non-canonical ubiquitination and arginylation (references are provided within each reported PTM).

**Mutation data**. Human mutation data, specifically ‘Substitution - Missense’ types, were obtained from the COSMIC database (38).

**E3 interactome data**. The interactomes were acquired from the BioGRID (39), IntAct (40), UbiNet 2.0 (41) databases and from the literature (11,26), focusing on E3 ligases interactions. E3 ligase annotations were sourced from the AMIGO web server (using query GO:00616300) (42), ESBL (43) and UbiNet 2.0.

**Proteolytic cleavage sites data**. The experimental proteolytic cleavage sites specific for a given protein with adherent information about the involved proteolytic enzymes were derived from the MEROPS database (44) and literature (references are provided within each reported cleavage site). The MEROPS dataset was subsequently filtered to contain only cleavage sites classified as physiologically relevant with present information about their exact position in the sequence.

#### Secondary structure and solvent accessibility

The secondary structure and solvent-accessible surface area of each residue are calculated by the server using the mkdssp software (45,46), with the latter being normalized to the relative solvent-accessibility (RSA) by the Sander method (47).

#### Evolutionary conservation score

For every available MSA, four different degron conservation scores are computed, reflecting the motif preservation throughout the MSA and its adjacent area (that can be adjusted), as illustrated in Supplementary Figure S1.

#### Disorder prediction

DEGRONOPEDIA offers two methods to predict disordered regions in proteins: (i) using pLDDT/LDDT (predicted Local Distance Difference Test/Local Distance Difference Test, respectively) values from AlphaFold2/RoseTTAFold (48) models for structure-containing queries and (ii) using IUPred3 software (49), applicable to all query types, which calculates a disorder score for each amino acid based on the query sequence. If choosing the former, a structure model from AlphaFold2/RoseTTAFold is required with a B-factor column in the PDB file filled with valid pLDDT or LDDT scores, respectively. Both methods have adjustable thresholds (default <70 for the former and >0.5 for the latter to consider a residue as disordered) for defining disordered residues on the server, and IDRs are identified as continuous regions meeting the set length.

#### Proteolysis prediction

Cleavage analysis of a query protein can be conducted based on a user-specified cleavage motif/site, MEROPS dataset, or through predictions made using the Pyteomics module (50). This module forecasts cleavage sites for 35 distinct proteolytic enzymes, implementing the cleavage prediction rules of the PeptideCutter Expasy web server (51).

#### Machine learning models development

**Figure 2.**
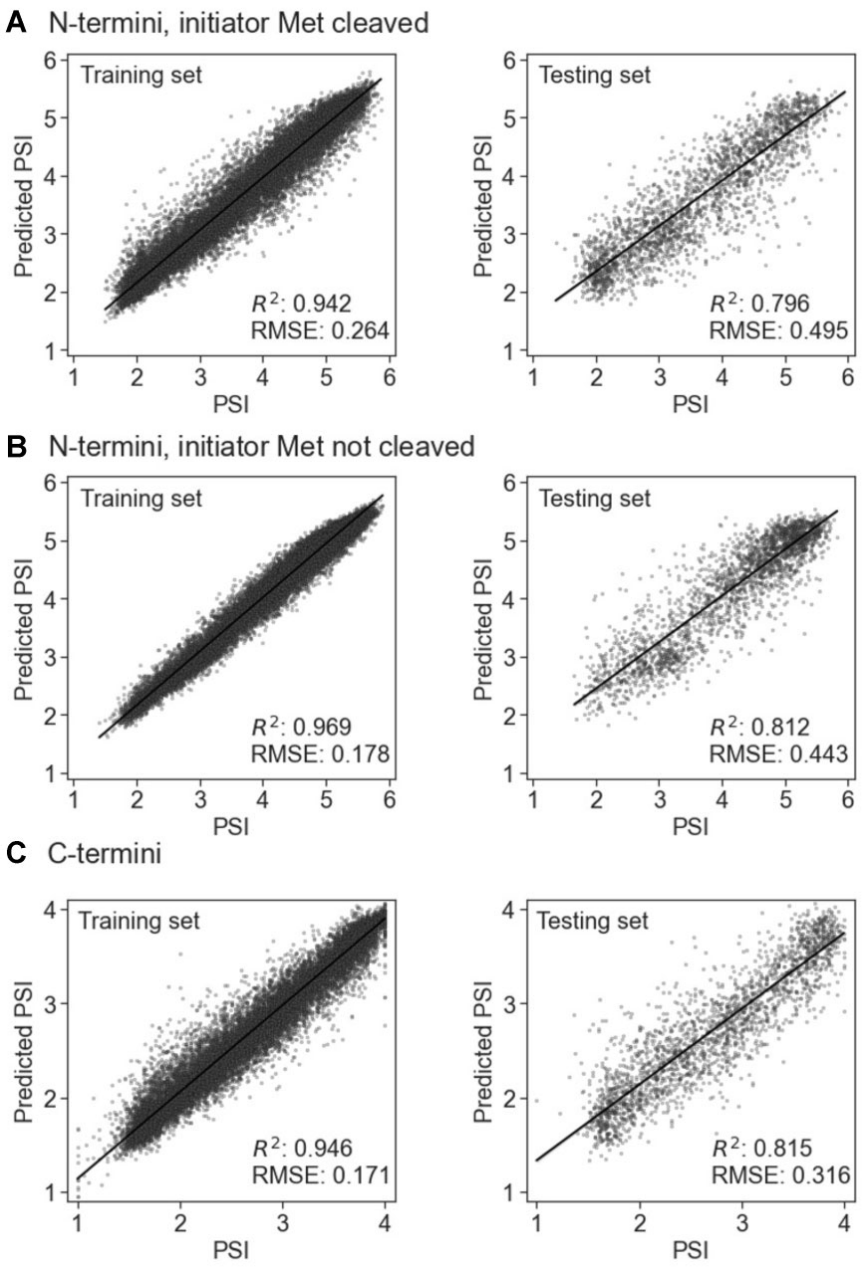
Predictions of the PSI using ML CatBoost regression models for N-termini with the initiator methionine (Met) cleaved (**A**) or not cleaved (**B**) and for C-termini (**C**). Scatter plots show a regression line with 95% confidence.

To develop our ML models, we used experimental N-/C-termini stability data, expressed as PSI values, from 23-mers covering the human proteome, considering both presence and absence of the initial methionine residue in N-termini. The datasets were divided into training and testing sets (90:10 ratio), and CatBoost regression models (52) were trained for each terminus (C-termini, N-termini with and without the initial methionine residue), optimizing hyperparameters with Optuna framework (53) and five-fold cross-validation (using random permutations cross-validation implemented in the scikit-learn Python library (54), with a 20% validation set). Descriptors included peptide sequences, RDKit descriptors (RDKit: Open-source cheminformatics. https://www.rdkit.org), Gravy hydrophobicity index, and the Peptides module (https://github.com/althonos/peptides.py). All descriptors were calculated for the whole sequence, and the first (excluding the N-terminal methionine regardless of whether we considered it as present or absent) or the last (for the C-terminus) ten, eight, six, four and two amino acids. Model performance was evaluated using a coefficient of determination (*R*^2^) and root mean square error (RMSE). The performance of all three models was similar, reaching *R*^2^ = 0.796 and RMSE = 0.495 for the N-terminus with initiator methionine cleaved, *R*^2^ = 0.812 and RMSE = 0.443 for the N-terminus with initiator methionine not cleaved, and *R*^2^ = 0.815 and RMSE = 0.316 for C-terminus (Figure 2). The server visualizes predicted PSI values, mapping them to experimental data distributions and classifying them into stability categories using quantile thresholds. Neptune management system was used for tracking ML experiments (neptune.ai: experiment tracker; https://neptune.ai).

#### Customizable parameters

Screening for known degron motifs is dynamically performed on the web server, considering ten parameters the user can customize on the homepage. In short, they describe thresholds related to the distinct regions of the tripartite degron architecture, classifying residues as buried or disordered and calculating evolutionary conservation (Supplementary Table S2).

#### Visualizations

Degrons visualizations in the sequence are created using FeatureViewer (55), while ProSeqViewer (56) and Logomaker (57) package are employed to display degrons within the MSAs. Graphics were created in BioRender.com.

### FBXL15 case study experimental approach

#### Cell culture

Flp-In 293 HEK293 cell line (female embryonic kidney epithelial cell line; ThermoFisher Scientific; authentication certificate issued by the manufacturer—checked for viability, mycoplasma, sterility and b-galactosidase activity) were cultivated in Dulbecco's Modified Eagle's Medium (Sigma) supplemented with 10% heat-inactivated Fetal Bovine Serum (Sigma) and 1% Antibiotic-Antimycotic (Gibco) at 37°C, 5% CO_2_ in a humidified incubator.

#### Plasmids

HiBiT fusion vectors were constructed by the sequence and ligation independent cloning (SLIC) method (58) using the parental vector pBiT3.1-N (Promega) or pBiT3.1-C (Promega) for the N- or C-terminal fusion variant, respectively, linearized with EcoRI (N-terminal variant) and SacI (C-terminal variant) enzymes, dephosphorylated and mixed with human FBXL15 sequence PCR-amplified from HEK293 cDNA (FBXL15 protein sequence corresponding to UniProt ID: Q9H469).

#### Cycloheximide chase assay

**Cells preparation and transfection**. HEK293 cells were seeded in white 96-well tissue culture plates (Greiner) at a density of 10.000 cells in a total volume of 100 μl per well. After 38–40 h, cells were transiently transfected with 2.5 ng HiBiT-tagged human FBXL15 expression constructs, diluted in carrier DNA (Promega) to obtain a final DNA amount of 50 ng/well. Transfection was carried out using the FuGENE HD Transfection Reagent (Promega) according to the manufacturer's guidelines in the Opti-MEM I Reduced-Serum Medium (Gibco), maintaining the 3:1 FuGENE HD:DNA ratio. Cells were incubated for 23 h at 37°C, 5% CO₂.

**Cycloheximide assay**. Cycloheximide (CHX; BioShop) was added to wells at a final 50 μg/ml concentration for 4 h. Treatment was performed in triplicate. Where indicated, cells were concomitantly treated with 20 μM MG132 (Selleck Chemicals) for 4 h. After a 4-h incubation with CHX, the Nano-Glo HiBiT Lytic Detection Assay (Promega) was performed according to the manufacturer's guidelines. Briefly, 120 μl of the Nano-Glo HiBiT Lytic Reagent was added to each well. Samples were mixed on an orbital shaker for 5 min and incubated for 10 min before luminescence measurement using TECAN Infinite 200 Pro plate reader equipped with the Magellan Pro software and an integration time of 1000 ms. The luminescence measurements were normalized in each well to the number of living cells (see below).

**Cell viability assay**. To assess cell viability during the CHX assays, they were multiplexed with the CellTiter-Fluor Cell Viability Assay (Promega) following the manufacturer's guidelines. Briefly, after 3-h incubation with CHX, 20 μl of the 5× concentrated CellTiter-Fluor Reagent was added to all wells. Cells were incubated at 37°C for 1 h, and fluorescence was measured using the Tecan Infinity M1000 fluorescence plate reader equipped with the Magellan Pro software with the parameters setup of 390nmEx/505nmEm. The untransfected cells were used as the global viability reference.

### SDE2 case study experimental approach

#### Cell culture

HEK293 cell line (ATCC; authentication certificate issued by the manufacturer—checked for viability, mycoplasma, sterility and b-galactosidase activity) were cultivated in Dulbecco's modified Eagle's medium (Sigma) supplemented with 10% heat-inactivated fetal bovine serum (Sigma) and 1% antibiotic–antimycotic (Gibco) at 37°C, 5% CO_2_ in a humidified incubator.

#### Plasmids

For transient transfection, the following were cloned into pCMV3 plasmid: HA-SDE2-FLAG (DU75320), HA-SDE2-FLAG G76,77A (DU75421), HA-SDE2-FLAG G76A (DU75344), HA-SDE2-FLAG G77A (DU75364), HA-SDE2-FLAG K78M (DU75354), HA-SDE2-FLAG K78A (DU75377). All constructs were sequence-verified by the DNA Sequencing Service, University of Dundee (https://www.dnaseq.co.uk). These constructs are available to request from the MRC PPU Reagents and Services webpage (http://mrcppureagents.dundee.ac.uk) and the unique identifier (DU) numbers provide direct links to the cloning strategies and sequence details.

#### Cycloheximide chase assay

**Cells preparation and transfection**. HEK293 cells were transferred in six-well plates with 6 × 10^5^ cells per well in 2 ml media in order to achieve 80% confluence the day after. Cells were transfected with 0.020 μg/μl plasmid solution in the Opti-MEM I Reduced-Serum Medium (Gibco) using FuGENE HD Transfection Reagent (Promega). All cells were lysed 48 h after transfection.

**Cycloheximide assay**. For the CHX chase experiment, 0.1% v/v of DMSO CHX (BioShop; 100 mg/ml final concentration) was added to the desired well at different time points—six, four, two, one and half hours before lysis. For the proteasome inhibitor treatment, MG132 (S2619, Selleck Chemicals) was added into the desired wells at 50 μM final concentration and 0.1% v/v of DMSO. DMSO (0.1% v/v final concentration) was added to the remaining wells as vehicle control. Cells were incubated for 4 h before harvesting. After incubation, the medium was removed, and cells were washed twice with ice-cold phosphate buffer saline. RIPA lysis buffer (Sigma) containing cOmplete protease inhibitor cocktail (Roche) was added, and the cells were collected by scraping. After the removal of the insoluble fraction by centrifugation, the protein concentration of the clarified lysate was determined by the Pierce Coomassie (Bradford) Protein Assay Kit.

**Immunoblotting**. Protein extracts were fractionated by SDS-PAGE on 4–12% Tris-acetate NuPage Novex (Life Technologies) polyacrylamide gels and transferred to a nitrocellulose membrane using wet transfer. The membrane was then blocked with 5% w/v bovine serum albumin in Tris-buffered saline with 0.1% w/v Tween-20. For detecting proteins, the following primary antibodies in the given concentrations were used: anti-GAPDH (Cell Signaling Technology, 14C10) 1:2000, anti-FLAG (Cell Signaling Technology, D6W5B) 1:1000, anti-HA (Cell Signaling Technology, C29F4) 1:1000. Following incubation with Licor 926-32211 IRDye 800CW Goat anti-Rabbit IgG (H + L), the signal was developed using Licor Odyssey CLx Imager.

## Results

### Workflow

**Figure 3.**
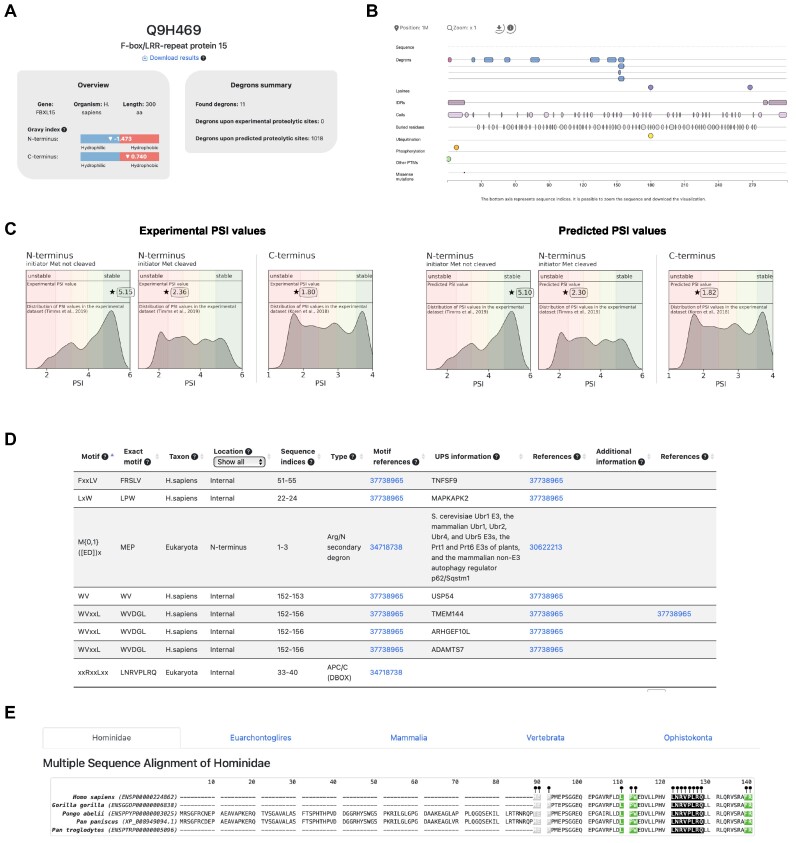
Overview of the DEGRONOPEDIA outputs using FBXL15 cullin-RING substrate receptor. (**A**) Overview panel with general information on the FBXL15 protein (left) and degrons summary panel with information on the number of found degron motifs (right). (**B**) Visualization of the found degron motifs, structural data, PTMs and mutations on the protein sequence. (**C**) Experimental (left) and ML-predicted (right) values of the N-/C-terminus of FBXL15 plotted on the distribution of experimental stability datasets. (**D**) Example of interactive output table providing information on the degrons found in the FBXL15. (**E**) Visualization of MSA with denoted positions of found degrons in the orthologs.

We outline the DEGRONOPEDIA workflow for queries using UniProt IDs, which offer the most exhaustive results (see Figure 1). Other query types follow a similar process but lack certain analyses due to limited access to experimental data. The workflow begins by screening the queried protein for degron motifs from our curated dataset, followed by calculating the Gravy hydrophobicity index (59) of the protein's terminal 15 amino acids, a step informed by the significant role hydrophobicity plays in N-/C-degron recognition by various E3 ubiquitin ligases (60,61). Subsequently, DEGRONOPEDIA offers experimental N-/C-terminus stability data, represented as PSI values (specifically for human proteins), sourced from GPS studies (11,12). It also facilitates predictions using our pre-trained ML models. Since these models were developed using human PSI values, we suggest their use primarily for queries related to higher mammals. The server then visualizes PSI values, categorizing them into stability classes; we also offer a standalone tool for high-throughput PSI prediction (available at https://github.com/filipsPL/degronopedia-ml-psi). Additionally, we have integrated pre-calculated MSAs and offer the option for users to submit custom MSAs, enabling the provision of evolutionary conservation scores derived from these alignments. Solvent-accessibility, location within an IDR region, or lack of secondary structure are premises of a site acting as an actual degron (15). Our server predicts IDRs and degrons’ disorder using AlphaFold2/RoseTTAFold models or IUPred3 software, depending on the input type, and calculates secondary structure and relative solvent-accessibility for each degron motif. As degron sites may undergo various PTMs, with the primary role of phosphorylation, which can modulate their exposure (62), the server reports experimentally validated PTMs (up to 32 types) occurring within each found degron motif and its flanking regions. Amino acid substitutions in degron motifs can lead to altered protein stability, contributing to severe diseases such as cancer (18) or neurodegeneration (63), indicating critical sites for proper protein function. Therefore, our tool provides information about known missense mutations within the degron motifs and within PTMs in their flanking regions. For each identified degron, the server also evaluates its context in the tripartite degron model. In particular, the server provides information on solvent accessibility, secondary structure, location within an IDR, mean disorder, PTMs, and mutations for each potentially ubiquitinated residue (secondary degron; in our implementation, these are not only lysines (K) but also cysteines (C), serines (S) or threonines (T)) located within the degron flanking regions. Finally, the server reports the closest IDR (tertiary degron) to each of the secondary degrons. It has been shown that protein turnover may be regulated by proteolytic enzymes that cleave the protein, leading to new N- and C-termini, which may act as degrons (5). Thus, DEGRONOPEDIA simulates protein cleavage based on user-defined motifs, validated cleavage sites, or predicted sites for various proteases, subsequently screening the cleavage products for degrons. The final information provided relates to E3 ligases known to interact with the queried protein. The occurrence of degrons in the sequence, along with their unique features, is visualized, and the outcomes are presented in easy-to-navigate interactive tables. The tutorial and introductory video, accessible from the web server, provide clear step-by-step instructions on the server functionality. The excerpt of outputs that DEGRONOPEDIA provides is illustrated in Figure 3.

### Computation times

Queries are processed relatively fast when degron conservation scores are not considered – depending on the submitted query type, the mean calculation time for a 393 aa p53 protein ranges from 25 to 27 s, for a 2549 aa mTOR from 42 to 45 s, and for a 5890 aa AHNAK from 78 to 90 s. The calculation of conservation scores requires longer times—88 s for p53, 180 s for mTOR, and 145 s for AHNAK. A comparison of computation times and different types of queries with varying input protein lengths can be found in Supplementary Table S3.

### Example application: validating ML-predicted degron in FBXL15

**Figure 4.**
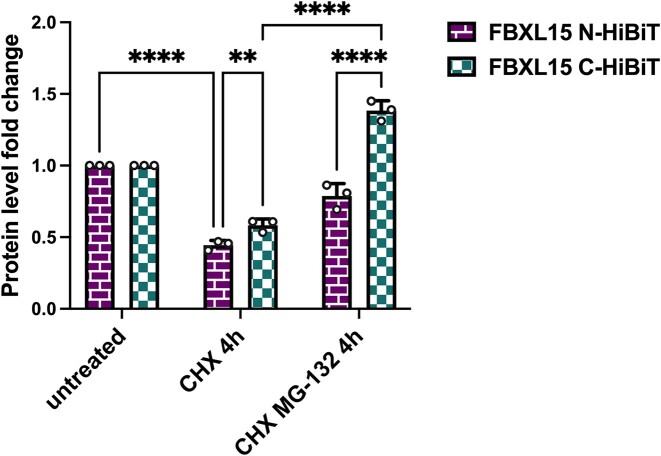
FBXL15 possess a destabilizing C-terminus. N- or C-terminus-HiBiT-tagged FBXL15 turnover was measured by CHX assay with transient expression of FBXL15 fusion variants and co-treatment with 50 μg/ml CHX for 4 h and, where indicated, 20 μM MG132 proteasome inhibitor. Protein levels were measured and normalized to the number of living cells as described in Materials and Methods; assays were normalized to the corresponding measurement for each protein variant from control time 0′. Error bars denote the standard deviation from the mean derived from three biological replicates; dots represent the biological replicates. Each biological replicate is a mean of three technical replicates. Data was analyzed using two-way ANOVA and the significance levels obtained from Tukey's multiple comparisons test are indicated for the compared conditions (∗∗∗∗ *P* ≤ 0.0001; ∗∗ *P* ≤ 0.01).

To ascertain the accuracy of DEGRONOPEDIA in predicting protein terminal stability, our study centered on human FBXL15 (UniProt ID: Q9H469), a cullin-RING ligase complex receptor (64), identified by the tool as having an unstable C-terminus (Figure 3). To validate these predictions, which are based on ML model that has been trained on stability data of C-terminal 23-mers, we employed Promega HiBiT system, a technique previously validated by our research (65) for its efficacy in examining the stability of other cullin receptors. We attached the HiBiT tag, comprising a short 11 amino acid sequence, to both the N- and C-termini of FBXL15, aiming to obstruct the protein's C-terminal degron and potentially modify its stability, in line with DEGRONOPEDIA projections. To assess changes in stability, we conducted cycloheximide (CHX) chase assays in HEK293 cells, a methodology integral to inhibiting protein synthesis and thereby allowing the tracking of FBXL15 degradation. The interaction between HiBiT and the complementary LgBiT peptide facilitated the precise quantification of FBXL15 levels through luminescence. Our findings revealed a notable increase in FBXL15 stability when the HiBiT tag was attached to its C-terminus, particularly after a 4-hour CHX treatment (Figure 4). This heightened stability contrasted with the lower stability observed in the case of the N-terminal HiBiT variant. Further, the application of the proteasome inhibitor MG132 led to significant accumulation of both protein variants, especially the C-terminally tagged version, indicating proteasome-dependent degradation of FBXL15. Of note, we chose FBXL15, as the exact experimental data of 23-mer C-terminal stability data were available for this protein, which confirms the reliability of our ML predictor and affirms the presumed instability of FBXL15 untagged C-terminus, pinpointing DEGRONOPEDIA accuracy in the studies on full-length proteins.

### Example application: verifying predicted degron in SDE2 post-proteolysis

**Figure 5.**
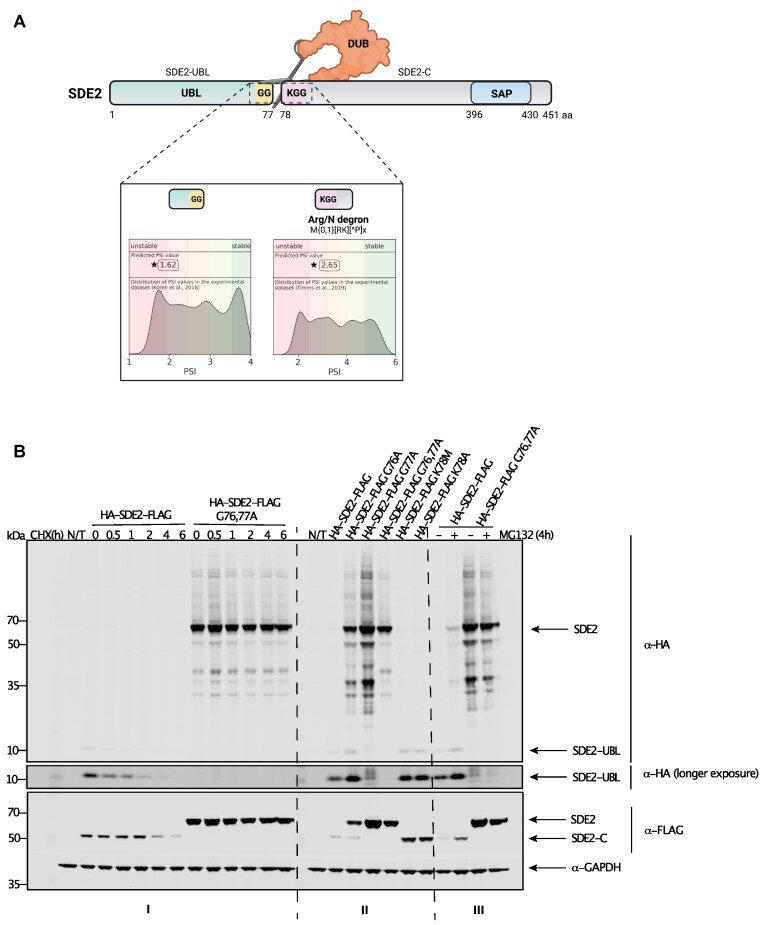
SDE2 proteolysis leading to new cleavage product degraded by Arg/N-degron pathway. (**A**) Schematic representation of SDE2 proteolysis occurring at the 77 amino acid position, leading to newly cleaved products. DEGRONOPEDIA-performed PSI predictions of the new termini. UBL – ubiquitin-like domain; DUB – deubiquitinating enzyme; SAP – DNA-binding SAP domain. (**B**) SDE2 stability depends on positive post-translational processing and degradation via the proteasome. I. CHX assay with transient expression of HA-SDE2-FLAG or G76,77A mutant treated. The levels of SDE2-C, SDE2-UBL and SDE2 (full-length) were determined by western blotting. II. HEK293 cells expressing indicated HA-SDE2-FLAG variants. The levels of SDE2-C, SDE2-UBL and SDE2 (full-length) were determined by western blotting. III. HEK293 cells expressing HA-SDE2-FLAG or G76,77A mutant treated with DMSO control (–) or proteasome inhibitor MG132 (+) for 4 h. The levels of SDE2-C, SDE2-UBL and SDE2 (full-length) were determined by western blotting. N/T – not treated.

To test DEGRONOPEDIA ability to identify and evaluate the stability of newly revealed N-termini of proteins that undergo proteolytic processing, we performed a case study on the splicing regulator SDE2 protein (UniProt ID: Q6IQ49). SDE2 plays critical roles in several cellular processes, including ribosomal biogenesis, telomere silencing, DNA replication, and pre-mRNA processing (66–68). SDE2 is expressed as a ubiquitin-fold precursor protein carrying an N-terminal ubiquitin-like domain. Proteolytic cleavage immediately downstream of the ubiquitin-like domain (SDE2-UBL) at the diglycine G76-G77 motif releases an activated fragment of the C-domain of SDE2 (SDE2-C). It was previously reported that destabilizing lysine 78 (K78) at the neo-N-terminus of SDE2-C directs it to proteasomal degradation (67). To verify if DEGRONOPEDIA would cope with simulating proteolytic cleavage by deubiquitinating enzymes (DUBs) and assessing the stability and presence of neo-N/C-terminus degrons, we analyzed human SDE2 in our tool. DEGRONOPEDIA indicated the previously reported processing site pointing at low stability of both SDE2 fragments (SDE2-UBL and SDE2-C) and an Arg/N primary degron in SDE2-C (Figure 5A). To confirm this, we expressed SDE2 tagged with HA (at N-terminus) and FLAG (at C-terminus) to monitor both fragments simultaneously using immunoblotting and anti-HA and anti-FLAG antibodies, respectively. The SDE2-C fragment was short-lived in the CHX chase assay, with a half-life of approximately 3 hours, in agreement with previous data (67). A double mutation of the G76-G77 cleavage site to alanine (G76,77A) completely abolished processing, resulting in a stable, full-length, non-cleavable SDE2. Importantly, mutations of K78 (K78M or K78A), which abolished the N-terminal degron, stabilized SDE2-C. To test whether the observed short half-life depended on active proteasomal degradation, cells overexpressing HA-SDE2-FLAG or G76,77A double mutant were treated with the proteasome inhibitor MG132 for 4 hours before harvesting. Proteasome inhibition led to increased levels of SDE2-UBL and SDE2-C fragments, consistent with the proteasome-dependent degradation expected for targets of the N-degron pathway (Figure 5B).

In the case studies featured, DEGRONOPEDIA demonstrates a unique capability absent in current methodologies: the prediction of N-/C-terminal degrons based on the most comprehensive GPS data, as well as the identification of degrons emerging from proteolysis. This platform uniquely positions itself among existing online tools, a comparison of which is detailed in Supplementary Table S4.

## Discussion

DEGRONOPEDIA aligns with Guharoy *et al.* tripartite degron model (15), offering an intuitive interface for comprehensive degron analysis. It accepts various input forms, including UniProt ID, FASTA sequences and PDB structures, with the depth of output tailored to the input type. The server excels in identifying known degron motifs, analyzing overlapping PTMs and mutations, and detecting novel N-/C-pathway degron motifs post-simulated proteolysis. It also evaluates evolutionary conservation scores and predicts protein terminal stability using ML models.

A key feature of DEGRONOPEDIA is its incorporation of PSI information, providing insights into terminal stability. While high PSI values do not preclude degron presence, low PSI values strongly suggest susceptibility to the N-/C-degron pathway. This is exemplified in our study on FBXL15 from the cullin-RING ligase complex, where DEGRONOPEDIA predictions of an unstable C-terminus were experimentally validated using the HiBiT system.

However, DEGRONOPEDIA has certain limitations. It does not support batch queries and can process sequences up to 40 000 amino acids or up to 5 MB file size for structure uploads. UniProt ID queries are currently restricted to proteins from 11 model organisms. For other taxa or non-reference isoforms, sequence or structure queries are the alternatives. Regarding disorder predictions, it is important to note that pLDDT/LDDT scores from AlphaFold2 models may not always align with disordered regions. Aderinwale *et al.* analysis revealed that only 30–50% of residues with low pLDDT scores matched disordered regions predicted by other methods (69). Hence, pLDDT scores should not be the sole metric for identifying IDRs. To address this, DEGRONOPEDIA allows users to adjust the pLDDT threshold for disorder or employ the IUPred3 tool for sequence-based predictions.

DEGRONOPEDIA also tackles the challenge of false positives in degron identification due to the highly degenerate nature of short linear motifs. The server enhances prediction accuracy by integrating additional contextual information, including secondary structure, RSA, IDRs, PTMs, mutations, E3 ligase interactions, and evolutionary conservation. This comprehensive approach aids in distinguishing functional degrons, offering a reliable starting point for experimental validation.

Future DEGRONOPEDIA updates will focus on improving IDR predictions, offering features for consecutive N-/C-terminus depletion analysis, and enhancing visualization through structure viewers. We plan to continually integrate new datasets to keep the server updated with the latest research developments. We also recognize the evolving understanding of degron architecture, transitioning from simple, linear motifs to multifaceted, non-linear arrangements that present significant challenges for existing predictive tools such as DEGRONOPEDIA. A substantial shift in our methodology is required to address this complexity, underscoring the critical demand for sophisticated computational techniques and comprehensive structural data to identify non-continuous degrons reliably.

## Supplementary Material

gkae238_Supplemental_File

## Data Availability

DEGRONOPEDIA is a comprehensive online web server available at https://degronopedia.com.
