# Supplementary material for: DEGRONOPEDIA: a web server for proteome-wide inspection of degrons
Source: Nucleic Acids Res. 2024 Apr 3;52(W1):W221–32. doi: 10.1093/nar/gkae238 (PMC11223883; doi:10.1093/nar/gkae238)
Supplement: gkae238_Supplemental_File [file gkae238_supplemental_file.pdf]

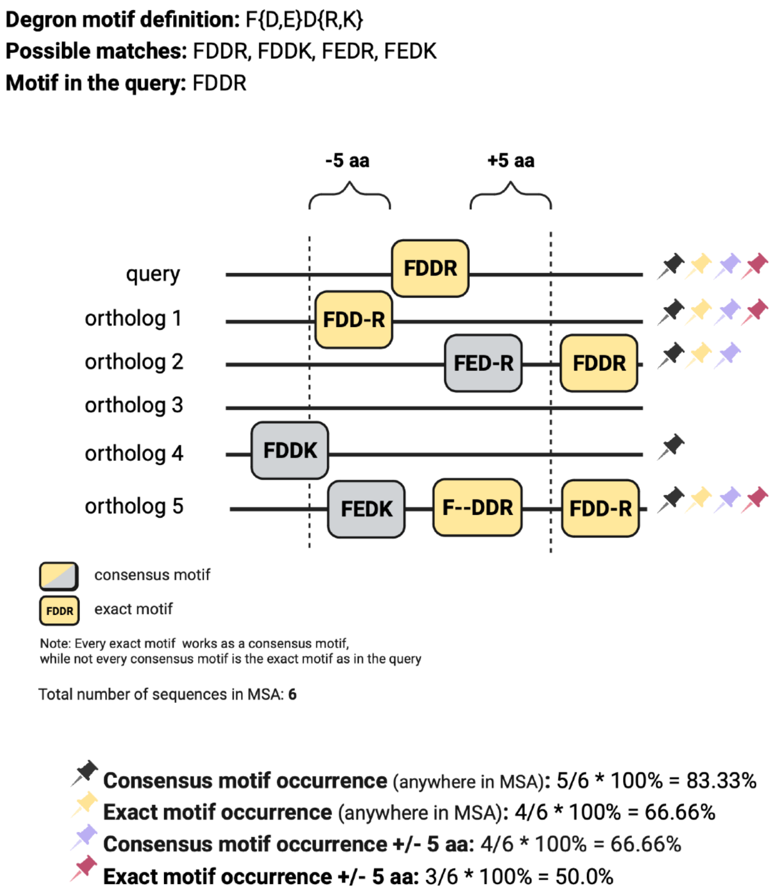

Figure S1. Four different conservation scores calculated by DEGRONOPEDIA. The server calculates the fraction of occurrences of the exact degron motif present in the query anywhere in the aligned sequences, as well as +/- 5 aa adjacent to the degron motif and the consensus motif (degrons are usually reported as a combination of consecutive amino acids), anywhere in the aligned sequences, and +/- 5 aa adjacent to the degron motif. The indicated threshold of +/- 5 aa in the query can be adjusted (see Customizable parameters in Materials and Methods).

Table S1. Pre-calculated MSAs of predicted orthologs at various taxonomic levels obtained from the eggNOG5 database available for selected model organisms. NCBI taxonomy identifiers are given in parentheses.

Species	Orthologous Group 1	Orthologous Group 2	Orthologous Group 3	Orthologous Group 4	Orthologous Group 5	Orthologous Group 6
H. sapiens	Hominidae (9604)	Euarchontoglires (314146)	Mammalia (40674)	Vertebrata (7742)	Opisthokonta (33154)	Eukaryota (2759)
M. musculus	Rodentia (9989)	Euarchontoglires (314146)	Mammalia (40674)	Vertebrata (7742)	Opisthokonta (33154)	Eukaryota (2759)
D. rerio	Actinopterygii (7898)			Vertebrata (7742)	Opisthokonta (33154)	Eukaryota (2759)
D. melanogaster	Drosophilidae (7214)	Diptera (7147)	Insecta (50557)		Opisthokonta (33154)	Eukaryota (2759)
C. elegans	Rhabditida (6236)				Opisthokonta (33154)	Eukaryota (2759)
S. cerevisiae	Saccharomycetaceae (4893)	Saccharomycetes (4891)	Ascomycota (4890)	Fungi (4751)	Opisthokonta (33154)	Eukaryota (2759)
A. thaliana	Brassicales (3699)		Viridiplantae (33090)			Eukaryota (2759)
S. pombe	Taphrinomycotina (451866)	Saccharomycetes (4891)	Ascomycota (4890)	Fungi (4751)	Opisthokonta (33154)	Eukaryota (2759)
O. sativa	Poales (38820)		Viridiplantae (33090)			Eukaryota (2759)
Z. mays	Poales (38820)		Viridiplantae (33090)			Eukaryota (2759)
R. norvegicus	Rodentia (9989)	Euarchontoglires (314146)	Mammalia (40674)	Vertebrata (7742)	Opisthokonta (33154)	Eukaryota (2759)

Table S2. Description of the user-customizable parameters available in DEGRONOPEDIA.

Parameter	Unit	Default value	Allowed values	Description
**Primary degron-related**				
1. Degron flanking region in sequence	aa	20	5-40	The maximum sequence distance to regions upstream and downstream of the degron motif to be considered as flanking
2. Degron flanking region in the structure	Å	20	5-40	The maximum structural distance to residues around the degron motif to be considered as flanking (such residues are not necessarily close in sequence to the degron motif)
3. Region length to calculate degron disorder	aa	10	1-20	The maximum sequence distance to regions upstream and downstream of the degron motif to be included in the degron mean disorder score
**Secondary degron-related**				
4. Region length to calculate secondary degron (K/C/T/S)[1] disorder	aa	3	1-15	The maximum sequence distance to regions upstream and downstream of the secondary degron (K/C/T/S) to be included in the secondary degron mean disorder score
**Tertiary degron-related**				
5. Minimum IDR distance from the secondary degron (K/C/T/S)	aa	10	5-40	The minimum sequence distance from the secondary degron (K/C/T/S) to the continuous IDR of defined length (see parameter 6) to consider it as a tertiary degron
**Structure-related**				
6. Minimum continuous IDR length	aa	10	5-40	The minimum number of subsequent (in sequence) disordered residues to be considered as IDR

7. pLDDT/LDDT disorder threshold	%	70	40-90	The threshold below which the residue is considered as disordered based on its confidence pLDDT/LDDT score; the structure for which its disorder is to be predicted based on the pLDDT/LDDT scores must be either an AlphaFold2 (default when querying by UniProt ID) or RoseTTAFold model
8. IUPred3 disorder threshold	%	50	40-90	The threshold above which the residue is considered disordered based on predictions obtained from the IUPred3 software (sequence-based predictions)
9. Buried residue threshold	%	20	5-60	The threshold below which the residue is considered as buried based on its RSA
**Multiple sequence alignment (MSA)-related**				
10. Maximum distance from the degron motif in the query to consider the same degron motif from the aligned sequences as conserved	aa	5	5-40	The maximum distance to regions upstream and downstream of the degron motif (its ends) in the query to its aligned sequences in the MSA to consider it as evolutionarily conserved

[1] lysine/cysteine/threonine/serine residues

Table S3. Comparison of computation times and different types of queries with varying input protein lengths. All calculations included machine learning predictions of N-/C-terminus stability and disorder predictions based on IUPred3 software; all other settings remained default and no optional parameters were provided. Queries by structure were run on structures corresponding to the indicated UniProt identifiers (in parentheses) obtained from the AlphaFold Protein Structure Database v.4.

Protein	Query by UniProt ID		Query by sequence	Query by structure
	Degron conservation ✗	Degron conservation[1] ✓		
**p53** – 393 aa (P04637)	~ 25 s	~ 88 s	~ 25 s	~ 27 s
**mTOR** – 2549 aa (P42345)	~ 42 s	~ 180 s	~ 42 s	~ 45 s
**AHNAK** – 5890 aa (Q09666)[2]	~ 90 s	~ 145 s	~ 78 s	~ 90 s

[1] When considering degron conservation, calculation time depends on the number of degron motifs found in the query, for which conservation is calculated based on the available Multiple Sequence Alignments (MSAs; also with varying number of member sequences). The conservation scores for p53 were calculated based on six different MSAs, while those for mTOR and AHNAK were calculated based on four.

[2] Since this protein sequence exceeded 2700 amino acids, an in-house script was used to merge the AlphaFold2 model fragments into a single PDB file.

Table S4. Comparison of functionalities of DEGRONOPEDIA and other online methods for degron analysis.

	**APC/C degron repository**	**ELM prediction tool**	**DegPred**	**DegronMD**	**DEGRONOPEDIA**
Detected/predicted degrons	ABBA, D box and KEN box degrons only	**32 degron motifs** (N-/C- or internal motifs)	Predicts/maps degrons based on **303 degron instances**	Predicts/maps degrons based on **23 degron motifs (133 degron instances)**	Maps N-/C- and internal degrons based on **over 1800 degrons** and **predicts N-/C-terminal degrons**
Screen by custom degron motifs	Yes	No	No	No	Yes
N-/C-terminal stability	No	No	No	No	Yes
Analysis of degrons emergence following proteolysis	No	No	No	No	Yes
Degron tripartite model	No	No	No	No	Yes
Query by protein name/UniProt ID	Yes	Yes	Yes	Yes	Yes
Query by protein sequence	No	Yes	No	Yes	Yes

Query by protein structure	No	No	No	No	Yes
Query by species other than human	Yes	Yes	No	No	Yes
Adjust parameters for degron screen	No*	No	No	No	Yes
MSA of homologs	Yes	Yes	No	Yes	Yes
Analyze own MSA	No	No	No	No	Yes
Degron conservation scores	No	Yes	No	Yes	Yes
Post-translational modifications	Yes	Limited to phosphorylation	No	Yes	Yes
Mutations related to human disease	Yes	No	No	Yes	Yes
Structural data	Yes	Yes	Yes	Yes	Yes
Interacting E3 ubiquitin ligases	No	No	Yes	Yes	Yes

* Cut-offs for disorder score and flank length may be adjusted when screening a proteome
